# HIV-1 drug mutations in children from northern Tanzania

**DOI:** 10.1093/jac/dku087

**Published:** 2014-04-11

**Authors:** Elichilia R. Shao, Emmanuel G. Kifaro, Innocent B. Chilumba, Balthazar M. Nyombi, Sikhulile Moyo, Simani Gaseitsiwe, Rosemary Musonda, Asgeir Johannessen, Gibson Kibiki, Max Essex

**Affiliations:** 1 Kilimanjaro Christian Medical Center, Moshi, Tanzania; 2 Kilimanjaro Christian Medical University College, Moshi, Tanzania; 3 Kilimanjaro Clinical Research Institute, Moshi, Tanzania; 4 Botswana Harvard HIV Research Laboratory, Gaborone, Botswana; 5 Vestre Viken HF Drammen Hospital, Drammen, Norway

**Keywords:** dried blood spots, antiretroviral therapy, mutations, sub-Saharan Africa, early infant diagnosis, child

## Abstract

**Objectives:**

In resource-limited settings, it is a challenge to get quality clinical specimens due to poor infrastructure for their collection, transportation, processing and storage. Using dried blood spots (DBS) might be an alternative to plasma for HIV-1 drug resistance testing in this setting. The objectives of this study were to determine mutations associated with antiretroviral resistance among children <18 months old born to HIV-1-infected mothers enrolled in prevention of mother-to-child transmission services in northern Tanzania.

**Patients and methods:**

Kilimanjaro Christian Medical Center (KCMC) Clinical Laboratory is the zonal centre for early infant diagnosis using DBS in northern Tanzania. DBS were collected from January 2011 to December 2012. Mothers were kept on triple therapy and single-dose nevirapine before pregnancy and during labour, respectively. Infants were given single-dose nevirapine and most of them were breastfed. Genotypic resistance was determined in those with a viral load of >400 copies/mL.

**Results:**

Genotypic resistance mutations were detected in 13 of 46 children (28%). HIV-1 genotypes were A1 (*n* = 27), C (*n* = 10), A/D (*n* = 4), D (*n* = 3) and CRF10_CD (*n* = 2). The median age was 12 weeks (IQR 6–28). The mean log_10_ viral load was 3.87 copies/mL (SD 0.995). All major mutations were detected in the reverse transcriptase gene and none in the protease gene region. The most frequent mutations were Y181C (*n* = 8) and K103N (*n* = 4), conferring resistance to non-nucleoside reverse transcriptase inhibitors.

**Conclusions:**

One-third of infants newly diagnosed with HIV in northern Tanzania harboured major drug resistance mutations to currently used antiretroviral regimens. These mutations were detected from DBS collected from the field and stored at room temperature. Surveillance of drug resistance among this population in resource-limited settings is warranted.

## Introduction

Globally, 3.4 million children were living with HIV at the end of 2011, of which 91% reside in sub-Saharan Africa. The majority acquired HIV from their HIV-infected mothers during pregnancy, birth or breastfeeding. Transmission of HIV from mother to child can be effectively prevented by timely provision of antiretroviral treatment (ART) to the mother.^1^ However, scaling up of prevention of mother-to-child transmission (PMTCT) services towards zero paediatric infection by 2015, as recommended by the WHO, comes with many challenges in low- and middle-income countries.^2^ Important obstacles to effective PMTCT are late detection of mothers in need of ART, lack of reliable HIV tests for infants, limited laboratory capacity to detect treatment failure and lack of paediatric antiretroviral formulations.^3^

Expansion of ART programmes in resource-limited settings has radically changed the face of the HIV/AIDS pandemic; however, there is an increasing need for surveillance of transmitted drug-resistant HIV.^4^ There are limited data on genotypic resistance results from DBS collected from the field environment in sub-Saharan Africa. So far, only two studies have been done in Tanzania using DBS to determine genotype data. No study has been conducted on ART drug resistance mutations obtained from DBS collected for the purpose of early infant diagnosis from children <18 months in the country.^5–7^ Furthermore, challenges in rural settings are more pronounced due to a shortage of well-trained health workers, cold chain facilities, transportation and other logistics of service deliveries.^8^ Therefore, we aimed to determine HIV drug resistance (HIVDR) in children <18 months of age born to HIV-1-infected mothers enrolled in PMTCT services using DBS in northern Tanzania.

## Patients and methods

From January 2011 to December 2012, a retrospective cross-sectional study was carried out among children <18 months old born to HIV-1-infected mothers. This is the group of children diagnosed during early infant diagnosis using DNA PCR technology to avoid false positive as a result of maternal antibodies. These exposed children were given single-dose nevirapine syrup as prophylaxis to protect them from maternal viral infection. Dried blood spots (DBS) with two or more saturated spots were considered eligible. Ethical clearance was sought and granted by the Kilimanjaro Christian Medical College review board followed by the Botswana–Harvard partnership for approval of the material transfer agreement. Mothers and caregivers provided consent before collection of DBS. We used 122 DBS cards from HIV-1-positive children collected from four regions, namely Kilimanjaro, Manyara, Arusha and Tanga. These DBS were collected according to guidelines from the National AIDS Control Programme. Positive PCR DBS were shipped from the Kilimanjaro Christian Medical University College Clinical Laboratory to the Botswana–Harvard Partnership HIV Research Laboratory for analysis.

RNA was extracted from two circles of DBS using the NucliSENS silica-based extraction method (bioMérieux, Durham, NC, USA) according to the manufacturer's instruction. DBS specimens for HIV viral load were analysed using NucliSENS EasyQ HIV-1 v2.0 Analyzer (bioMérieux, Canada) with a lower detection limit of 20 copies/mL. Specimens with a viral load of >400 copies/mL were subjected to RT–PCR followed by nested PCR. We amplified the entire protease (PR) region and a portion of the reverse transcriptase (RT) region, representing 1.6 kb of HIV-1 *pol*. Sequencing was done using a 16 capillary 3130 XL ABI Prism Genetic Analyzer sequencer (Applied Biosystems, Foster City, Canada). Sequencher version 5.0 premier DNA software was used to edit raw sequences manually and form a contig in fasta format file, which was then submitted to the Stanford HIV Drug Resistance Database for analysis. To determine HIV-1 subtypes, the REGA HIV-1 and HIV-2 automated subtyping tool version 2.0 was used to assign all subjects with pure HIV-1 subtype as it differs from other inter subtypes. The PR and RT for subtyping and drug resistance-associated mutations were interpreted using the Stanford Genotypic Resistance Interpretation Algorithm for specimens collected from HIV-1-infected infants and the Calibrated Population Resistance tool was used.^6^ The sequences obtained were submitted to GenBank under accession numbers KJ482111–KJ482156. The main outcomes of interest were HIV-1 subtype diversity and major drug resistance mutations to currently used regimens in the region. The data were analysed using SPSS version 16.0 for Windows (SPSS Inc., Chicago, IL, USA). All tests were two-sided and the level of significance was set at *P* < 0.05.

## Results

Out of 640 tested children, 122 had a positive HIV DNA test and were included in the study. Out of these, 22 (18.03%) DBS were excluded due to fungal contamination, while 37 (30.32%) were excluded due to viral load <400 copies/mL. Twelve (9.84%) failed amplification and 5 (4.10%) had an undetectable viral load. The mean log_10_ viral load was 3.87 copies/mL (SD 0.995). The remaining 46 (37.70%) were eligible for subtyping and genotypic testing.

In total, 13 of 46 (28%) children successfully genotyped exhibited major drug resistance-associated mutations in the RT gene, of whom 12 had resistance to non-nucleoside reverse transcriptase inhibitors (NNRTIs), while one had resistance to both nucleoside reverse transcriptase inhibitors (NRTIs) and NNRTIs (Table 1). The most frequent RT mutation was Y181C (*n* = 8), which causes high-level resistance to all NNRTIs. K103N (*n* = 4) was the second most common RT mutation and causes high-level resistance to nevirapine and efavirenz, but not to the newer NNRTIs etravirine and rilpivirine. Y188L and G190A, which cause high-level resistance to NNRTIs, were detected in two children. M184V was detected in one child who was on nevirapine single-dose syrup prophylaxis. This mutation is specific for lamivudine-containing regimens and confers resistance to lamivudine and emtricitabine. Another interesting finding was a strain with the Y181C mutation in a sample collected from a child born to an HIV-1-infected mother; the mother and child were not kept on any ART regimen, though they were enrolled with the antenatal services. Table 2 shows the subjects with detected drug resistance-associated mutations and the relation to ART prophylaxis and breastfeeding. Of concern is one virus strain with multiclass resistance: Y188L for NNRTIs and M184V for NRTIs. We also found a case of a transitional mutation, T215S, which is detected in isolates that eventually go on to develop the NRTI drug resistance mutations T215Y and T215F.

**Table 1.** Subjects with at least one HIVDR mutation among children <18 months of age from northern Tanzania

ID	Viral load (copies/mL)	NRTI resistance-associated mutations	NNRTI resistance-associated mutations	Affected drugs
1	8700		**K103N**	NVP, EFV
2^a^	20 000	T215S		
3	9400	T69D	V106I	
4	820 000	V179T		
5	5700		**K103N**	NVP, EFV
6	8300		**Y181C**	ETR
7	38 000		**Y181C**	NVP
8	8300		**Y181C**	NVP
9	1 200 000		**Y181C**, **G190A**	EFV, NVP
10^b^	130 000	**M184V**	**Y188L**	NVP, EFV, RPV, 3TC, FTC
11	1400	A71T	**K103N**, **Y181C**	NVP, EFV
12	4000		**Y181C**	NVP
13	99 000		**K103N**	NVP, EFV
14	200 000	V118I		
15	1500		**Y181C**	NVP
16	38 000		**Y181C**	NVP
17^b^	47 000	T69K, **V75I**/V	V106I, **Y181C**	NVP

EFV, efavirenz; ETR, etravirine; FTC, emtricitabine; 3TC, lamivudine; NVP, nevirapine; RPV, rilpivirine.

All major mutations according to the Stanford HIV Database are in bold, whereas underlined mutations are minor mutations. We have also reported transitional mutations: T215S.

^a^Participant with a transitional mutation, i.e. any mutation that by itself does not cause resistance but that indicates evolving resistance, e.g. RT mutation T215S can be detected in isolates that go on to develop drug resistance mutations T215Y and T215F.^20^

^b^Participant having dual-class resistance.

**Table 2.** Presence of at least one major HIVDR mutation among children infected with different HIV-1 subtypes in northern Tanzania

ID	HIV-1 subtype	Major mutation(s)	Pre-labour prophylaxis for mothers	Labour prophylaxis for mothers	Infant prophylaxis	Mode of feeding
1	A	K103N	none	sdNVP	sdNVP	exclusively breastfed
2	A	K103N	ZDV	none	sdNVP	exclusively breastfed
3	A	Y181C	none	none	sdNVP	alternative feeding
4	A	Y181C	ZDV	none	sdNVP	exclusively breastfed
5	A	Y181C	none	sdNVP	sdNVP	alternative feeding
6	A	Y181C	ZDV	sdNVP	sdNVP	exclusively breastfed
7	A	K103N	none	none	sdNVP	alternative feeding
8	A	Y181C	ZDV	cART	sdNVP	exclusively breastfed
9	A	Y181C, V75I	none	none	none	mixed feeding
10	A	Y181C, G190A	none	sdNVP	sdNVP	mixed feeding
11	C	M184V, Y188L	cART	cART	sdNVP	exclusively breastfed
12	C	K103N, Y181C	none	cART	none	exclusively breastfed
13	D	Y181C	ZDV	cART	sdNVP	exclusively breastfed

cART, combination antiretroviral treatment, i.e. zidovudine (ZDV) + lamivudine + nevirapine.

sdNVP, single-dose nevirapine, as per Tanzania regimen protocol.^5^

Major drug resistance means mutations that by themselves reduce susceptibility to one or more drugs.^20^

## Discussion

The results from this study demonstrate that nearly one-third of the transmitted HIV-1 strains had major mutations associated with antiretroviral drug resistance. At the end of 2013 WHO recommends provision of lifelong ART for all HIV-positive pregnant and breastfeeding women in order to prevent mother to child transmission of HIV, this is known as option B+. In the current study we showed that NNRTI-associated mutations were predominant, which means that option A and B for PMTCT (in which only women with CD4+ T cells <350 cells/mm^3^ or those with advanced disease were given lifelong ART), despite reducing vertical transmission of HIV, may lead to the development of drug-resistant HIV. NNRTI-associated mutations were predominant, showing that option A and B for PMTCT, despite a reduction in the vertical transmission of HIV, may lead to the development of drug-resistant HIV. We excluded many samples because they were not of good quality according to WHO due to poor storage conditions, including room temperature in the area with sunlight exposure.^9^ Of the exclusions, 18% were due to fungal contamination as all samples were stored at room temperature for 6 months. A few samples yielded very short sequences which might be caused by degradation of nucleic material as they were at room temperature from collection time to processing time.

The Y181C mutation was detected in two-thirds of the cases and this mutation causes high-level resistance to nevirapine, 2-fold decreased susceptibility to efavirenz and 5-fold decreased susceptibility to etravirine and rilpivirine.^9^ K103N was the second most predominant mutation, conferring high-level resistance to nevirapine and efavirenz. We detected two mutations, Y188L and G190A, in addition to other NNRTI-associated mutations, which cause high-level resistance to nevirapine, efavirenz and rilpivirine. Since NNRTIs are the most widely used first-line drugs in resource-limited settings, these children are at risk of treatment failure and premature death.^10^

Our findings are in line with a meta-analysis by Arrive *et al*.,^10^ who found NNRTI mutations among 52.6% of HIV-1-exposed infants who became infected from mothers who were enrolled in PMTCT B services. Similar studies from Uganda and Malawi, among infants infected after single-dose nevirapine, showed resistance to NNRTI in 50% and 80%, respectively.^11^ The most recent WHO recommendations for PMTCT recommend option B+, whereas plan A and B, which were used in the current study, are no longer supported due to the high risk of developing resistance.^9,11^

The mutation M184V was detected in one child who was not on a regimen containing lamivudine. The mutation was detected in a child who was breastfed by a mother on combination ART containing lamivudine, proving the transmission of an HIV-1-resistant strain because this infant was not on lamivudine. This mutation causes high-level resistance to lamivudine and emtricitabine.^12^ Another interesting finding was a strain with the Y181C mutation from a child born to an HIV-1-infected mother, both being antiretroviral naive. This indicates the occurrence of natural resistance mutations among drug-naive individuals, as reported previously in Kilimanjaro. It might result from a transmitted HIV-1 drug-resistant strain or undisclosed use of ART from the black market.^13,14^ We also detected one virus strain with dual-class resistance: Y188L for NNRTIs and M184V for NRTIs. The detection of isolates with T215S in our cohort indicates the presence of drug selective pressure and strongly suggests resistant viruses.^12^

The minor mutations found in the PR are consistent with naturally occurring non-B subtype polymorphisms. The absence of protease inhibitor resistance in our cohort supports the use of a protease inhibitor-based ART regimen in HIV-infected children recently exposed to PMTCT, as recommended by the WHO.^13^

Our study has certain limitations. First, the study was small, which means that the estimates of the proportion of children with drug resistance might be inexact. Second, the prevalence of drug resistance in this study might be underestimated because there is a tendency of fading away from detection with increasing time from ART exposure.^15,16^

With standard population sequencing we might have missed some of the resistant strains, because in the absence of ART the wild-type virus might outgrow the resistant strains.^17–19^ Finally, the failure to achieve an HIV-1 RNA sequence in 46 of 122 children might have skewed our results; however, it is unlikely that this was a systematic error affecting our major conclusions.

In summary, we found that drug resistance was common among children in a routine PMTCT programme in northern Tanzania. The regimens containing nevirapine, used as a prophylaxis for both mothers and infants, put infants at high risk of subsequent treatment failure. The challenges of using DBS in resource-limited settings include collection, transportation and storage conditions in combination with a shortage of healthcare workers and the long distance to the zonal laboratory, which might contribute to poor performance. DBS might be very useful in replacing plasma if WHO protocols for collection and storage are followed strictly. Our results indicate that there is a need for surveillance of drug resistance mutations among newly infected children as well as for improving DBS storage conditions in Tanzania.

## Funding

This work was supported by collaborative funds from Fogarty HIV Research, Training Health Researchers into Vocational Excellence in East Africa (THRiVE), (grant number 087540) funded by the Wellcome Trust, Tanzania, Zambia and Botswana consortia (TanZamBo) and the Kilimanjaro Clinical Research Institute (KCRI).

## Transparency declarations

None to declare.

## Disclaimers

The findings and conclusions in this report are those of the authors and do not necessarily represent the views of the funders.

Use of the trade names is for identification only and does not constitute endorsement by the funders.
